# Meta-analysis of clinical efficacy of thoracoscopy and robotic surgery in the treatment of mediastinal tumors

**DOI:** 10.1186/s12957-024-03325-5

**Published:** 2024-02-28

**Authors:** Jiying Dang, Shize Sun, Zhengqi Wu, Yidong Shan, Huiling Zhang

**Affiliations:** Wuwei Liangzhou Hospital, Gansu, China

**Keywords:** Video-assisted thoracic surgery, Robot-assisted thoracic surgery, Mediastinal tumors, Meta-analysis

## Abstract

**Objective:**

Comparing the clinical efficacy of thoracoscopy and robotic surgery in the treatment of mediastinal tumors using meta-analysis.

**Methods:**

Computer retrieval of PubMed, Embase, The Cochrane Library, and Web of Science databases for literature comparing the clinical effects of video-assisted thoracic surgery (VATS) and robot-assisted thoracic surgery (RATS) in treating mediastinal tumors, with the retrieval time limit from the establishment of the database to September 2023. Two evaluators independently screened the literature, extracted data, and assessed the risk of bias. Meta-analysis was performed using RevMan 5.4.

**Results:**

A total of 19 articles were included, with a total of 3517 patients. The results of the Meta-analysis showed that the RATS group had less intraoperative bleeding [MD =  − 5.20, 95%CI (− 9.28, − 1.12), *P* = 0.01], lower rate of conversion to thoracotomy [OR = 0.41, 95%CI (0.23, 0.72), *P* = 0.002], lower rate of total postoperative complications [OR = 0.57, 95%CI (0.34, 0.95), *P* = 0.03], shorter postoperative drainage time [MD =  − 0.72, 95%CI (− 1.13, − 0.32), *P* = 0.0004], and shorter postoperative hospital stay [MD =  − 0.90, 95%CI (− 1.16, − 0.65), *P* < 0.001], in comparison with the VATS group. There was an insignificant difference between the two groups in terms of tumor size [MD =  − 0.02, 95%CI (− 0.33, 0.30), *P* = 0.91] and operation time [MD = 0.17, 95%CI (− 7.61, 7.94), *P* = 0.97]. However, in regards to hospitalization costs [MD = 2634.75, 95%CI (991.62, 4277.88), *P* = 0.002], the RATS group was more expensive than the VATS group.

**Conclusion:**

Robot-assisted mediastinal tumor resection surgery has more advantages in terms of intraoperative bleeding, conversion to thoracotomy rate, total postoperative complication rate, postoperative drainage time, and postoperative hospital stay, in comparison with thoracoscopic-assisted mediastinal tumor resection surgery. There is an insignificant difference in tumor size and operation time between the two surgeries. However, robot-assisted mediastinal tumor resection surgery increases hospitalization costs.

## Introduction

Mediastinal tumors are common diseases in thoracic surgery, including thymoma, neurogenic tumors, teratomas, etc. [1, 2]. Clinically, surgery is generally the first choice of treatment, and most patients have achieved good prognosis and improved quality of life after treatment [3]. Compared with the classic median sternotomy or anterolateral intercostal incision thoracotomy approach, a large number of cases have proven that minimally invasive surgery has advantages such as small incisions, minor trauma, fewer complications, and faster postoperative recovery [4]. Minimally invasive surgery is now widely used in the treatment of mediastinal diseases. And thus, minimally invasive surgery has been extensively employed to treat mediastinal diseases. Nevertheless, the traditional thoracoscopy has some inherent shortcomings, such as two-dimensional depth of field, insufficient handling ability in small spaces (especially in the upper mediastinum and pleural apex lesions), and difficulties in operations such as suturing and knotting [5]. In view of the defects of video-assisted thoracic surgery (VATS), robot-assisted thoracic surgery was developed. The advantages of the robot system are that it can provide a high-resolution 3D field of view up to 10–15 times magnification, and thus realize a clearer observation of the structural details in the mediastinum [6]. At the same time, robot-assisted thoracic surgery (RATS) can completely filter out the physiological tremors of the human hand to avoid accidental injury due to misoperation [7].

Previously available investigations have revealed the feasibility and safety of VATS or RATS in the treatment of mediastinal tumors. However, all of the previous results belong to single-center retrospective studies with small sample sizes. In the treatment of mediastinal tumors, it is still unclear whether RATS can achieve the same or even better surgical results as VATS. Therefore, our present work incorporates the latest literature and conducts a comprehensive meta-analysis in order to provide a higher level of evidence-based medical evidence for clinical practice.

## Materials and methods

This meta-analysis was performed by the Preferred Reporting Items for Systematic Reviews and Meta-Analyses (PRISMA) guidelines and was registered in PROSPERO (CRD42023468350).

### Eligibility criteria

Inclusion criteria: (i) included study types: retrospective studies, prospective studies, or randomized controlled trials; (ii) study subjects: patients diagnosed with mediastinal tumors and meet the surgical indications; (iii) intervention measurements: RATS or VATS treatment of mediastinal tumors; (iv) outcome indicators: operation time, intraoperative blood loss, conversion to thoracotomy rate, postoperative drainage time, postoperative hospital stay, total postoperative complication rate, etc.

Exclusion criteria: (i) literature with incomplete data; (ii) non-clinical comparative studies such as reviews, case reports, experience summaries, or single-arm efficacy observations; (iii) repeatedly published literature.

### Literature search methods

Using a combination of subject words and free words for retrieval, the computer searches the PubMed, Embase, The Cochrane Library, and Web of Science databases for literature comparing the clinical effects of RATS and VATS in the treatment of mediastinal tumors. The retrieval time limit is from the establishment of the database to September 2023. The main English search terms are robot-assisted thoracic surgery, robotic, robot-assisted, da Vinci, video-assisted thoracic surgery, video-assisted, mediastinal tumors, mediastinal neoplasms, mediastinum cancers, and mediastinal cancer.

### Screening and data extraction of literature

Two researchers independently completed the literature search and assessed whether to preliminarily include the study by reading the titles and abstracts of the retrieved literature. After the preliminary search was completed, they read the full text of the retrieved literature to decide whether to include it in the study. For studies with doubts, the decision on whether to include them was made after discussion with the corresponding author of this study.

### Quality evaluation of the included studies

The Newcastle–Ottawa Scale (NOS) is used to score the included studies, which includes a total of 9 items, including the selection of cohorts (4 points), comparability (2 points), and exposure (3 points) [8].

### Statistical analysis

Use RevMan 5.4 software for statistical analysis. Statistical consolidation and quantitative evaluation of continuous variables are performed by calculating the mean difference (MD), and the 95% confidence interval (CI) of the MD is also calculated. Use the odds ratio (OR) for statistical consolidation and quantitative evaluation of binary variables, and calculate the 95% CI of the OR. The heterogeneity between study results is calculated using the default Q test method of RevMan 5.4 software to calculate chi^2^ and *I*^2^. If *I*^2^ < 50%, *P* > 0.1, it is considered that there is insignificant heterogeneity between the studies, and the data are analyzed using a fixed-effects model. If *I*^2^ > 50%, *P* ≤ 0.1, it is considered that there is significant heterogeneity between the studies, and the data is analyzed using a random-effects model. *P* ≤ 0.05 indicates that the difference is statistically significant. Sensitivity analysis is used to test the stability of the results, and funnel plots are used to judge publication bias.

## Results

### Literature search results

**Fig. 1:**
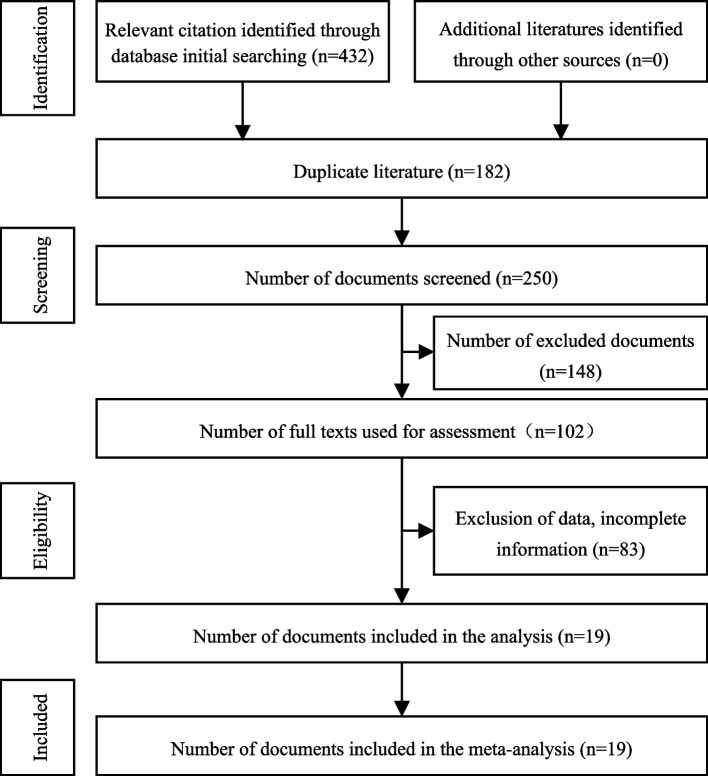
Flow diagram of literature retrieval and screening

A total of 432 articles were retrieved through the database. Finally, by reading the titles and abstracts, 182 duplicate articles and 250 obviously irrelevant articles were excluded; see Fig. 1. After careful reading, a total of 19 articles [9–27] were finally included. The detailed information and quality evaluation of the characteristics of all included studies are summarized in Table 1. A total of 3517 patients with mediastinal tumors were included, of which 1742 patients underwent RATS treatment and 1775 patients underwent VATS treatment.

**Table 1:** Basic information about the included studies

First author, Year	Study date	Country	Group	Patients	Male/female	Age (years)	NOS score
Jens 2011 [9]	1994–2006	USA	RATS	74	32/42	39(7–75)	8
			VATS	79	23/56	37(11–74)	
Ye 2013 [10]	2009–2012	China	RATS	21	13/12	53.4 ± 5.4	7
			VATS	25	9/12	52.7 ± 7.8	
Yi 2014 [11]	2009–2012	China	RATS	55	25/30	41.4(16–65)	8
			VATS	60	30/30	43.5(18–66)	
Rowse 2015 [12]	1995–2015	USA	RATS	11	6/5	52.2(23–74)	8
			VATS	45	19/26	50.6(23–87)	
Suda 2016 [13]	2011–2015	Japan	RATS	7	4/3	55.5 ± 9.9	8
			VATS	18	12/6	53.4 ± 14.8	
Qian 2017 [14]	2009–2014	China	RATS	51	21/30	48.8 ± 13.3	7
			VATS	35	19/16	50.3 ± 13.1	
Kamel 2019 [15]	2010–2014	USA	RATS	300	51/249	63	7
			VATS	280	50/230	62	
Abidin 2020 [16]	2010–2018	USA	RATS	21	13/8	41.29 ± 7.05	8
			VATS	24	14/10	42.52 ± 7.45	
Yang 2020 [17]	2010–2014	USA	RATS	77	31/46	60.9 ± 10.7	7
			VATS	77	32/45	61.1 ± 12.2	
Li 2020 [18]	2009–2014	China	RATS	60	30/30	53.72 ± 13.11	7
			VATS	60	30/30	51.22 ± 12.21	
Ali 2021 [19]	2006–2019	Denmark	RATS	39	32/7	58(20.5)	8
			VATS	13	9/4	68(13.0)	
Raja 2021 [20]	2009 ~ 2019	USA	RATS	380	154/226	43.2 ± 16.7	8
			VATS	340	129/211	40.6 ± 16.8	
Imielski 2021 [21]	2007 ~ 2017	USA	RATS	54	29/25	44.9 ± 15.8	8
			VATS	97	42/55	47.4 ± 15.2	
Salfity 2021 [22]	2010 ~ 2015	USA	RATS	325	151/174	63(22 ~ 90)	7
			VATS	263	120/143	64(20 ~ 88)	
Chiba 2022 [23]	2011 ~ 2021	Japan	RATS	20	7/13	55(34 ~ 88)	7
			VATS	37	16/21	61(30 ~ 82)	
Li 2022 [24]	2018 ~ 2021	China	RATS	106	45/61	46(34 ~ 57)	8
			VATS	106	45/61	48(40 ~ 56)	
Jiang 2022 [25]	2016 ~ 2022	China	RATS	61	40/21	46.10 ± 14.10	7
			VATS	36	19/17	47.60 ± 14.60	
Hong 2023 [26]	2014 ~ 2022	China	RATS	34	14/20	41.03 ± 7.38	8
			VATS	36	17/19	43.06 ± 6.82	
Ochi 2023 [27]	2014 ~ 2022	Japan	RATS	46	28/18	67(28 ~ 78)	8
			VATS	144	84/60	52.5(10 ~ 83)	

### Quality evaluation of the included literature

The NOS scores for the included cohort studies are shown in Table 1 and all studies were of high quality with NOS scores between 7 and 9.

### Meta-analysis results

#### Comparison of tumor size

**Fig. 2:**
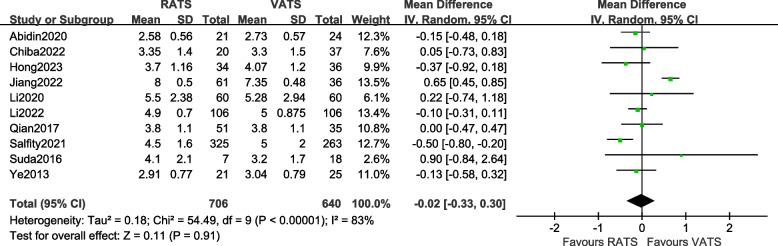
Meta-analysis forest plot of the tumor size

A total of 10 articles [10, 13, 14, 16, 18, 22–26] reported the tumor size of the two groups of patients, involving a total of 1346 patients. There was statistical heterogeneity between the studies (*P* < 0.00001, *I*^2^ = 83%, so a random-effects model was used for data analysis. The results [MD =  − 0.02, 95%CI (− 0.33, 0.30), *P* = 0.91] indicate that there is an insignificant difference in tumor size between the RATS group and the VATS group. The meta-analysis results are shown in Fig. 2.

#### Comparison of operation time

**Fig. 3:**
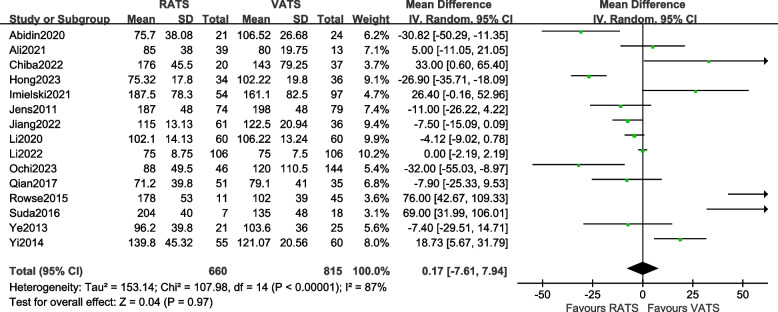
Meta-analysis forest plot of the operation time

A total of 15 articles [9–14, 16, 18, 19, 21, 23–27] reported the operation time of the two groups of patients, involving a total of 1475 patients. There was statistical heterogeneity between the studies (*P* < 0.00001, *I*^2^ = 87%, so a random-effects model was used for data analysis. The results [MD = 0.17, 95%CI (–7.61, 7.94), *P* = 0.97] indicate that there is a statistically insignificant difference in the operation time between the two surgical methods, suggesting that there is an insignificant difference in the comparison of operation time between the RATS group and the VATS group. The meta-analysis results are shown in Fig. 3.

#### Comparison of the incidence of intraoperative blood loss

**Fig. 4:**
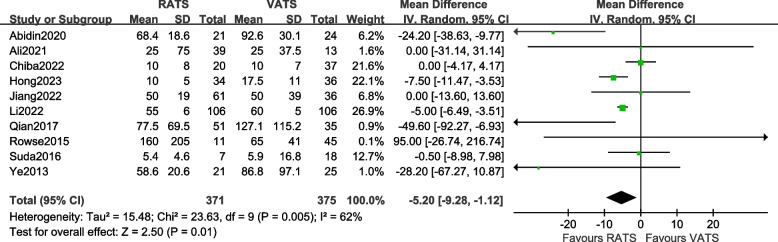
Meta-analysis forest of the intraoperative blood loss

A total of 10 articles [10, 12–14, 16, 19, 23–26] reported the intraoperative blood loss of the two groups of patients, involving a total of 746 patients. There was statistical heterogeneity between the studies (*P* = 0.005, *I*^2^ = 62%, so a random-effects model was used for data analysis. The results [MD = –5.20, 95%CI (− 9.28, − 1.12), *P* = 0.01] indicate that there is a statistically significant difference in intraoperative blood loss between the two surgical methods, suggesting that the RATS group had less intraoperative blood loss than the VATS group. The meta-analysis results are shown in Fig. 4.

#### Comparison of conversion to thoracotomy rate

**Fig. 5:**
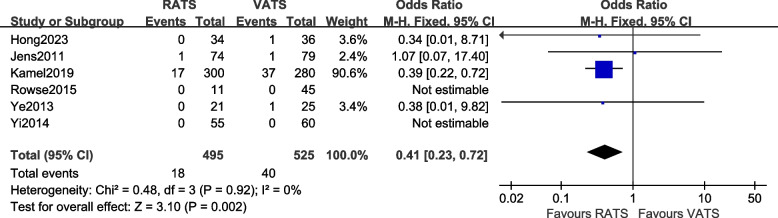
Meta-analysis forest plot of the conversion to thoracotomy rate

A total of 6 articles [10, 12–14, 16, 19, 23–26] reported the thoracotomy rate of the two groups of patients, involving a total of 1020 patients. There was no statistical heterogeneity between the studies (*P* = 0.92 > 0.1, *I*^2^ = 0%, so a fixed-effects model was used for data analysis. The results [OR = 0.41, 95%CI (0.23, 0.72), *P* = 0.002] indicate that there is a statistically significant difference in the conversion to thoracotomy between the two surgical methods, suggesting that the incidence of conversion to thoracotomy in the RATS group was lower than that in the VATS group. The meta-analysis results are shown in Fig. 5.

#### Comparison of incidence of the total postoperative complications

**Fig. 6:**
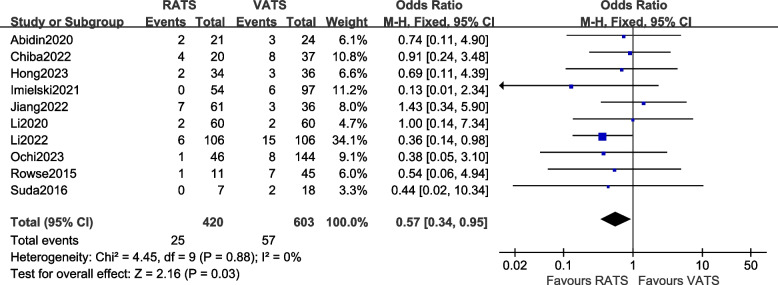
Meta-analysis forest plot of incidence of the total postoperative complications

A total of 10 articles [12, 13, 16, 18, 21, 23–27] reported the total postoperative complications of the two groups of patients, involving a total of 1023 patients. There was no statistical heterogeneity between the studies (*P* = 0.88 > 0.1, *I*^2^ = 0%, so a fixed-effects model was used for data analysis. The results [OR = 0.57, 95%CI (0.34, 0.95), *P* = 0.03] indicate that there is a statistically significant difference in the incidence of postoperative complications between the two surgical methods, suggesting that the incidence of postoperative complications in the RATS group was lower than that in the VATS group. The meta-analysis results are shown in Fig. 6.

#### Comparison of the incidence of postoperative drainage time

**Fig. 7:**
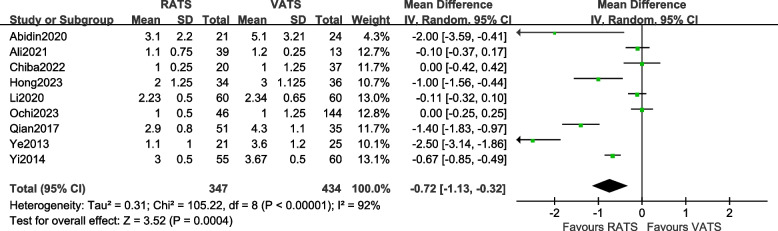
Meta-analysis forest plot of postoperative drainage time

A total of 9 articles [10, 11, 14, 16, 18, 19, 23, 26, 27] reported the postoperative drainage time of the two groups of patients, involving a total of 781 patients. There was statistical heterogeneity between the studies (*P* < 0.00001, *I*^2^ = 92%, so a random-effects model was used for data analysis. The results [MD =  − 0.72, 95%CI (− 1.13, − 0.32), *P* = 0.0004] indicate that there is a statistically significant difference in the postoperative drainage time between the two surgical methods, suggesting that the postoperative drainage time in the RATS group was shorter than that in the VATS group. The meta-analysis results are shown in Fig. 7.

#### Comparison of postoperative hospital stay

**Fig. 8:**
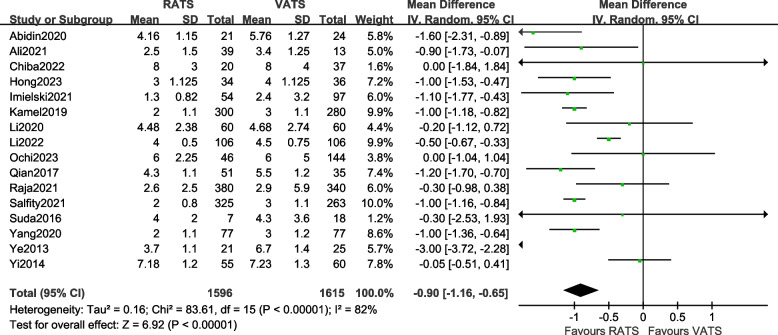
Meta-analysis forest plot of postoperative hospital stay

A total of 16 articles [10, 11, 13–24, 26, 27] reported the postoperative hospital stay of the two groups of patients, involving a total of 3211 patients. There was statistical heterogeneity between the studies (*P* < 0.00001, *I*^2^ = 82%, so a random-effects model was used for data analysis. The results [MD =  − 0.90, 95%CI (− 1.16, − 0.65), *P* < 0.001] indicate that there is a statistically significant difference in the postoperative hospital stay between the two surgical methods, suggesting that the postoperative hospital stay in the RATS group was shorter than that in the VATS group. The meta-analysis results are shown in Fig. 8.

#### Comparison of hospitalization costs

**Fig. 9:**
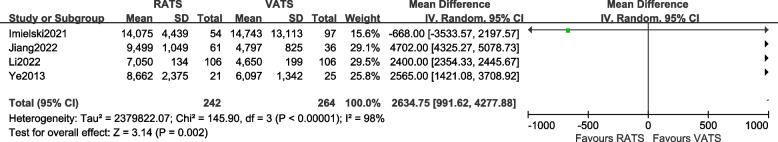
Meta-analysis forest plot of hospitalization costs

A total of 4 articles [10, 21, 24, 25] reported the hospitalization costs of the two groups of patients, involving a total of 506 patients. There was significant statistical heterogeneity between the studies (*P* < 0.00001, *I*^2^ = 98%, so a random-effects model was used for data analysis. The results [MD = 2634.75, 95%CI (991.62, 4277.88), *P* = 0.002] indicate that there is a statistically significant difference in hospitalization costs between the two surgical methods, suggesting that the hospitalization costs of the RATS group were higher than those of the VATS group. The meta-analysis results are shown in Fig. 9.

### Sensitivity analysis

Sensitivity analysis was performed on each outcome indicator using the one-by-one deletion method. The results of the sensitivity analysis showed that the effect size results did not change after the above indicators were deleted one by one, indicating that the stability of the results statistically combined after the above indicators is very high.

### Publication bias

**Fig. 10:**
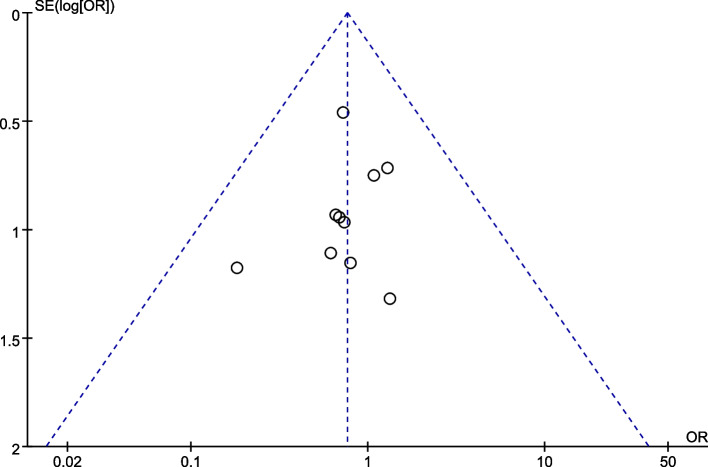
Funnel plot of incidence of the total postoperative complications

By drawing funnel plots for each outcome indicator, it was found that all studies were evenly distributed on both sides of the funnel plot, indicating that the publication bias of this study is small. The funnel plot drawn for the incidence of total postoperative complications as an example is shown in Fig. 10.

## Discussion

The mediastinal space is narrow, the structure is complex, the tissue origin is diverse, and it is adjacent to important organs such as large blood vessels and the heart [28, 29]. The current treatment principle for mediastinal tumors is still comprehensive treatment mainly based on surgery [28, 29]. In 2001, Yoskino performed the world’s first robot-assisted mediastinal tumor resection, laying the foundation for surgical robots to perform precise surgery in the narrow mediastinal area [30]. Currently, minimally invasive surgery is rapidly developing, and da Vinci robot-assisted mediastinal tumor resection and thoracoscopy-assisted mediastinal tumor resection are increasingly chosen by surgeons. However, whether robot assistance is superior to thoracoscopy assistance remains controversial. Therefore, we conducted a meta-analysis to explore and compare the efficacy and safety of RATS and VATS in the treatment of mediastinal tumors.

The results of the meta-analysis show that there is an insignificant difference in tumor size between the two groups. There is also an insignificant difference in operation time between the RATS group and the VATS group. According to previous research [11], the operation time of RATS is usually longer, which may be attributed to the additional setup time and the impact of the learning curve of RATS. With the accumulation of the surgeon’s experience and the proficiency of the robot team, the operation time of RATS can be significantly shortened. In terms of intraoperative blood loss, the results of this study show that the intraoperative blood loss in the RATS group is less than that in the VATS group. This probably stems from the fact that the RATS can provide a three-dimensional magnified view during surgery, more flexible operation and eliminate hand tremors, thereby accurately exposing the complex anatomical structure around the resection target [6]. This helps doctors to precisely perform operations during surgery and better control bleeding from small blood vessels.

In terms of conversion to thoracotomy, the RATS group had more advantages than the VATS group. Some of the conversions to thoracotomy are due to tumor invasion into the anonymous vein or pleural adhesion; another part of the conversion to thoracotomy is due to the large size of the tumor and suspected pericardial infiltration. In these cases, the choice of conversion to thoracotomy is reasonable. However, it should be noted that although the conversion rate to thoracotomy in the RATS group is lower than that in the VATS group, the conversion to thoracotomy in RATS is not as convenient as in VATS.

The occurrence of postoperative complications is an important indicator for assessing short-term postoperative efficacy. The results of this study show that the overall incidence of postoperative complications in the RATS group is lower than that in the VATS group. The main reasons are listed as follows: (1) compared with VATS, RATS recovers faster after surgery, and thus the risk of complications is significantly reduced. (2) RATS improves the comfort of the surgeon’s operation, which helps to increase the complete resection rate of tumors, the thoroughness of anterior mediastinal fat clearance, etc., on the basis of adhering to safety, tumor-free and minimally invasive, and reduces postoperative complications. In clinical practice, we have also found that RATS has more advantages in the anatomy of anterior mediastinal fat and adipose tissue. RATS can provide more detailed tumor anatomy, thereby better protecting the integrity of the tumor membrane.

In terms of postoperative drainage time, the RATS group had a shorter postoperative drainage time than the VATS group. We analyzed the results of the study and believed that this is related to the greater minimally invasive advantage of RATS than VATS. RATS makes the surgical process more precise, the hemostasis more thorough, and the stimulation to the surrounding tissues less. At the same time, the lesions are completely removed, and the surrounding adipose tissue can also be completely removed. All of the above-mentioned factors make the postoperative drainage time of the RATS group shorter. In terms of postoperative hospital stay, our research results disclosed that patients in the RATS group had a shorter postoperative hospital stay than the patients in the VATS group. This results from the fact that RATS is more minimally invasive, thus causing less postoperative pain and faster recovery. Patients recover faster after surgery, which shortens the postoperative hospital stay to a certain extent. This is also in line with the concept of enhanced recovery after surgery [31].

The limitations of this meta-analysis: (i) there is significant heterogeneity in hospital costs, which may be caused by differences in RATS charging standards in different medical centers; (ii) almost all of the studies included are retrospective clinical studies, selection bias is inevitable, and verification is needed with larger sample randomized controlled trials; (iii) there is currently a lack of long-term follow-up data for two types of surgeries, and we hope to further improve this data in the future; (iv) this study did not make a more detailed comparison of the location of mediastinal tumors, which may lead to some bias in the results.

## Conclusion

In summary, through the meta-analysis of the included literatures, we found that robot-assisted mediastinal tumor resection has more advantages in terms of intraoperative blood loss, conversion to thoracotomy rate, total postoperative complication rate, postoperative drainage time, and postoperative hospital stay than thoracoscopic-assisted mediastinal tumor resection. However, robot-assisted mediastinal tumor resection increases hospital costs. However, there is currently a lack of long-term follow-up studies on patients after surgery. We look forward to publishing more large-sample and high-quality randomized controlled studies in the future.

## Data Availability

No datasets were generated or analysed during the current study.

## Abbreviations

RATS: Robot-assisted thoracic surgery
VATS: Video-assisted thoracic surgery
NOS: Newcastle-Ottawa Scale
OR: Odds ratio
MD: Mean difference
CI: Confidence interval
